# Synchronous hepatocellular carcinoma and IPNB-associated intrahepatic cholangiocarcinoma in a patient with resolved viral hepatitis: a case report of diagnostic pitfall and margin-negative resection

**DOI:** 10.3389/fonc.2026.1894631

**Published:** 2026-10-28

**Authors:** Tong Zhou, Shuqing Wu, Tianze Guo, Weihua Zhu, Chen Wang, Jie Gao

**Affiliations:** 1 Department of Hepatobiliary Surgery, Peking University People’s Hospital, Beijing, China; 2 Peking University Research Center for Diagnosis and Treatment of Liver Cancer, Beijing, China; 3 Peking University Institute of Organ Transplantation, Beijing, China; 4 Department of Pathology, Peking University People’s Hospital, Beijing, China

**Keywords:** Brisbane 2000 classification, case report, hepatic sectionectomy, hepatocellular carcinoma, intraductal papillary neoplasm of the bile duct, intrahepatic cholangiocarcinoma, margin-negative resection

## Abstract

**Background:**

Synchronous hepatocellular carcinoma (HCC) and intraductal papillary neoplasm of the bile duct (IPNB)-associated intrahepatic cholangiocarcinoma (ICC) is a rare dual-lineage hepatobiliary malignancy. Although radical resection for conventional synchronous HCC and ICC has been well documented, reported experience with margin-negative (R0) resection for this specific tumor complex remains scarce.

**Case Summary:**

A 77-year-old male with resolved hepatitis B virus (HBV) infection and hepatitis C virus (HCV) infection with sustained virological response (SVR) presented with intermittent right upper quadrant pain, abdominal distension, scleral icterus, pruritus, nausea, and vomiting. Imaging studies revealed multifocal hepatic lesions and a biliary tract mass, and he was initially misdiagnosed as metastatic cholangiocarcinoma. Staging laparoscopy confirmed resectability, followed by open margin-negative resection. Intraoperative cholangioscopy and frozen-section analysis identified a pedunculated IPNB arising from the right hepatic duct and confirmed negative bile duct margins. Final pathology confirmed well-differentiated HCC and a separate high-grade IPNB with associated moderately to poorly differentiated ICC. The HCC and biliary lesion were separated by non-neoplastic parenchyma without a transition between them, excluding combined hepatocellular-cholangiocarcinoma (cHCC-CCA). Regional lymph nodes were not removed or histologically examined (pNx for both tumors). Margins exceeded 1 cm for both components. The patient recovered uneventfully; at 3-month follow-up, tumor markers were normal with no radiological recurrence.

**Conclusion:**

This report presents a rare case of margin-negative (R0) resection with unassessed nodal status (pNx) for synchronous HCC and IPNB-associated ICC in a patient with resolved HBV infection and HCV infection with SVR. The coexistence of multifocal hepatic lesions with a biliary tract mass should not be presumed to indicate metastatic disease; in this case, surgical exploration with intraoperative frozen-section analysis and cholangioscopy was useful for diagnosis and management, with the 3-month follow-up documenting only an early postoperative outcome.

## Introduction

The coexistence of multifocal hepatic lesions and a biliary tract mass is frequently misinterpreted as metastatic cholangiocarcinoma, often precluding curative-intent surgery. Synchronous hepatocellular carcinoma (HCC) and intrahepatic cholangiocarcinoma (ICC) constitutes the most frequently reported dual-lineage primary hepatic malignancy (1); however, the synchronous triad of HCC, intraductal papillary neoplasm of the bile duct (IPNB), and IPNB-associated ICC remains exceptionally rare, having been documented solely in a single autopsy case without attempted resection (2). Both hepatitis B virus (HBV) and hepatitis C virus (HCV) are established etiological agents in hepatobiliary carcinogenesis (3–6). Herein, we report what is, to our knowledge, the first case of margin-negative (R0) resection for this entity in a patient with resolved HBV infection and HCV infection with sustained virological response (SVR), who was preoperatively misdiagnosed as having metastatic cholangiocarcinoma. This case underscores that multifocal hepatic lesions with a biliary mass should not be reflexively interpreted as metastatic disease, and that surgical exploration with intraoperative pathological guidance supported diagnosis and margin-negative resection in this patient.

## Case presentation

A 77-year-old male with resolved HBV infection (HBsAg-negative, anti-HBc and anti-HBe positive) and HCV infection with sustained virological response (SVR) following 12-week sofosbuvir/velpatasvir therapy (completed 3 years prior) presented with intermittent right upper quadrant pain and abdominal distension for >6 months, acutely exacerbated over the preceding 2 weeks with scleral icterus, pruritus, nausea, and vomiting. He had no family history of malignancy or liver disease. He was a retired office worker with no history of smoking or occupational chemical exposure. He denied alcohol abuse, illicit drug use, and autoimmune liver disease.

On admission, physical examination was notable only for scleral icterus, with an otherwise unremarkable abdominal examination. Laboratory evaluation revealed elevated protein induced by vitamin K absence or antagonist-II (PIVKA-II) at 52.3 ng/mL, whereas alpha-fetoprotein (AFP; 4.35 ng/mL), carbohydrate antigen 19-9 (CA19-9; 9.4 U/mL), and carcinoembryonic antigen (CEA; 1.85 ng/mL) were within normal limits. Mild cholestasis was evident (γ-glutamyl transferase (GGT) 98 U/L, total bilirubin 24.4 μmol/L, direct bilirubin 15.9 μmol/L, albumin 37 g/L), with normal transaminases (alanine aminotransferase [ALT] 19 U/L, aspartate aminotransferase [AST] 24 U/L). His Eastern Cooperative Oncology Group (ECOG) performance status was 1. Comorbidities included hypertension, managed with regular oral amlodipine besylate and irbesartan. Liver function was preserved, corresponding to Child-Pugh class A (6 points) and a MELD score of 8 (preoperative INR, 0.96; serum creatinine, 83 μmol/L). Serological testing confirmed resolved HBV infection, with negative HBsAg (0.00 IU/mL; reference range 0–0.05), negative anti-HBs (1.99 mIU/mL; reference range 0–10), negative HBeAg (0.39 S/CO; reference range 0–1), positive anti-HBe (0.84 S/CO; values above 1 defined as negative), and positive anti-HBc (5.6 S/CO; reference range 0–1); serum HBV DNA was undetectable immediately before surgery. HCV RNA remained undetectable immediately before surgery, approximately 3 years after completion of antiviral therapy, consistent with sustained virological response.

Contrast-enhanced abdominal CT demonstrated hypodense lesions in hepatic segments VII and VIII (largest measuring 4.2 × 3.7 cm) with heterogeneous arterial-phase enhancement and persistent portal venous-phase enhancement (**Figures 1A–C**). Gadoxetic acid-enhanced liver-specific MRI showed the corresponding lesions (**Figure 2A**), with no hepatobiliary-phase uptake reported on the preoperative MRI examination. The largest imaging dimension (4.2 × 3.7 cm) corresponded to the segment VIII lesion, which measured 3.5 × 3.5 × 2 cm on final pathology; radiological and pathological measurements are reported separately because they were obtained using different methods. The segment VII lesion measured 3 × 2 × 2 cm on final pathology. Thickened bile duct walls with soft tissue nodules were observed in the proximal common bile duct and hilar bile ducts, accompanied by dilatation of the right hepatic duct and mild intrahepatic biliary dilatation (**Figures 1D-F**, **2B**). Preoperative future liver remnant volume was 57% of the total liver volume, and indocyanine green retention rate at 15 minutes (ICGR15) was 8.8%. On the basis of these imaging findings, the initial working diagnosis was metastatic cholangiocarcinoma with multifocal intrahepatic spread.

**Figure 1:**
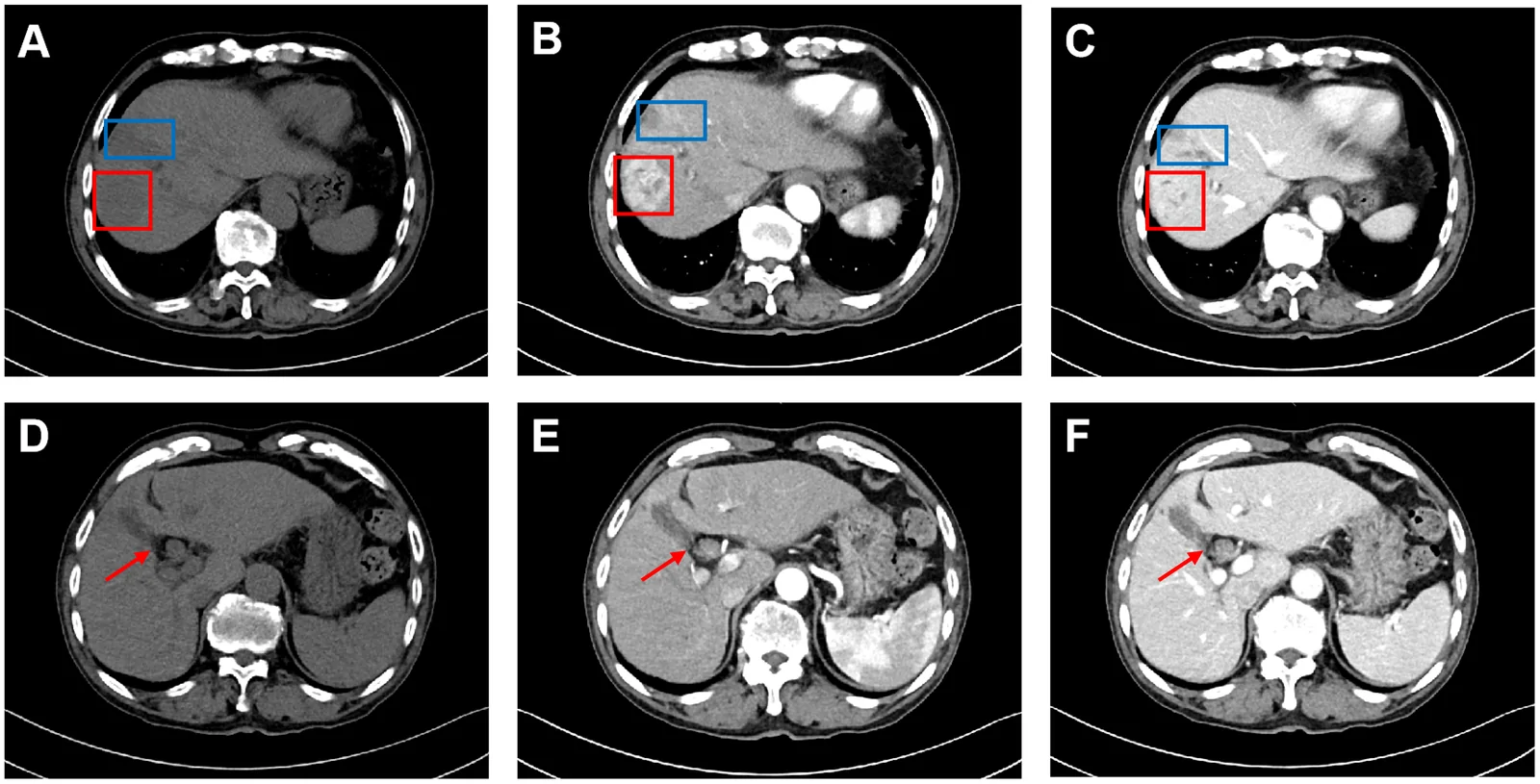
Preoperative unenhanced and contrast-enhanced abdominal computed tomography (CT). **(A–C)** Axial images showing the segment VII lesion (red boxes) and the segment VIII lesion (blue boxes), with heterogeneous arterial-phase enhancement and persistent portal venous-phase enhancement. The largest lesion, in segment VIII, measured 4.2 × 3.7 cm on preoperative imaging. **(D–F)** Axial images showing the dilated right hepatic duct (red arrows), with associated bile duct wall thickening. **(A, D)** Unenhanced images; **(B, E)** arterial-phase images; **(C, F)** portal venous-phase images.

**Figure 2:**
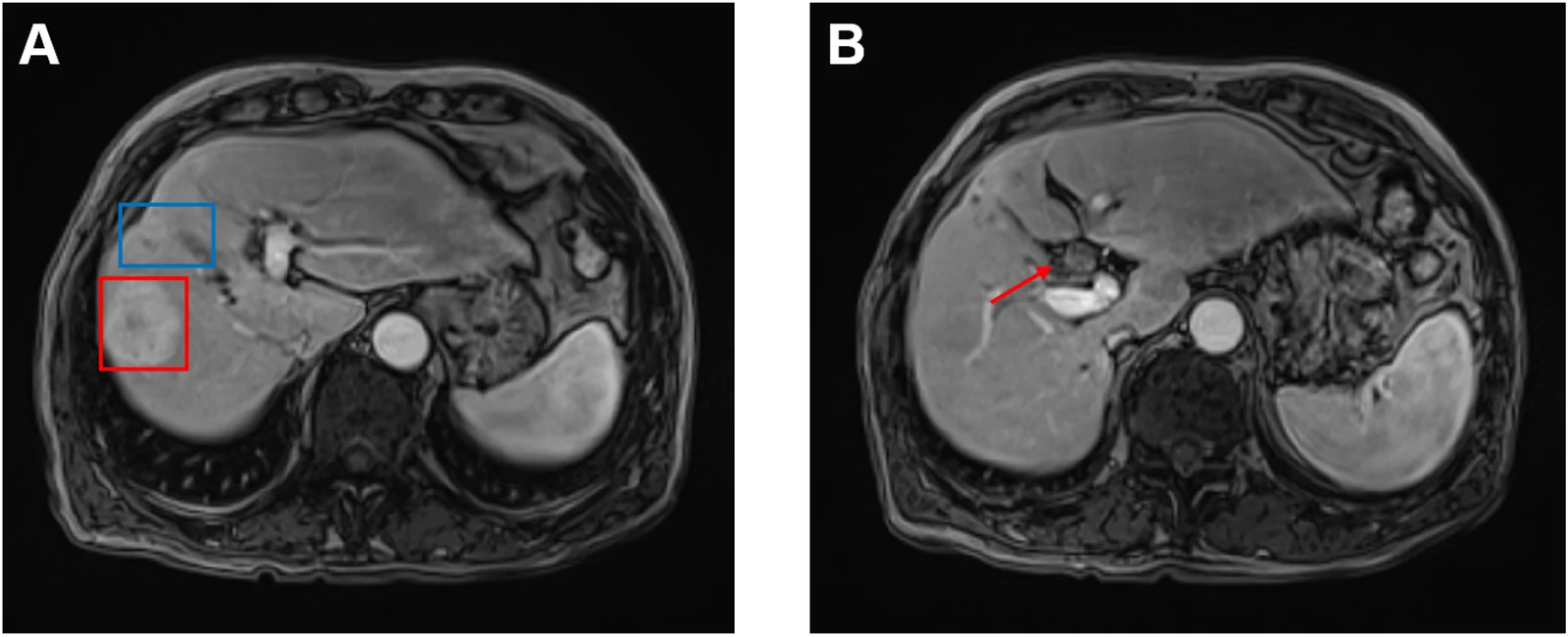
Gadoxetic acid–enhanced liver-specific magnetic resonance imaging. **(A)** Axial T1-weighted portal venous-phase contrast-enhanced image showing the segment VII lesion (red box) and the segment VIII lesion (blue box). **(B)** Axial T1-weighted portal venous-phase contrast-enhanced image showing the dilated right hepatic duct (red arrow) and mild intrahepatic biliary dilatation.

The case was discussed preoperatively at the institutional multidisciplinary tumor board (MDT). Preoperative biopsy of the biliary lesion (percutaneous or endoscopic) was considered but not performed because of the risks of biopsy-tract seeding and cholangitis, and because a tissue diagnosis would not have altered the decision to explore a potentially resectable lesion. Regional lymph node dissection was also discussed at the MDT; given the absence of enlarged or suspicious nodes on preoperative imaging, formal lymphadenectomy was planned only if suspicious nodes were encountered intraoperatively. Given the hilar location of the biliary mass and preoperative suspicion of multifocal disease, staging laparoscopy was initially performed to exclude radiologically occult peritoneal dissemination, thereby avoiding a non-therapeutic laparotomy in this 77-year-old patient. Laparoscopic inspection confirmed no peritoneal metastases or unresectable local extension. Intraoperative visual estimates identified a soft 2.5-cm mass on the surface of segment VII and a 4-cm firm whitish subcapsular mass in segment VIII. The procedure was converted to laparotomy. The patient subsequently underwent right anterior sectionectomy (segments V and VIII) combined with segment VII segmentectomy, cholecystectomy, hepatic ductoplasty, and T-tube drainage. This resection is equivalent anatomically to a right hepatectomy sparing segment VI, and was selected to preserve functional volume in a 77-year-old patient with resolved viral hepatitis. Hepatic inflow occlusion was performed three times using the Pringle maneuver (15 minutes each, separated by 5-minute intervals; total ischemic time 45 minutes). Intraoperative cholangioscopy confirmed a pedunculated IPNB arising from the right hepatic duct, with tissue protruding through the orifice of the right anterior hepatic duct. Proximal and distal bile duct margins were assessed by frozen-section examination. Regional lymphadenectomy was omitted given the patient’s advanced age, the absence of radiologically or intraoperatively suspicious lymph nodes, and the technical complexity of hilar biliary reconstruction necessitating minimization of operative time. The hilar, hepatoduodenal ligament, and peripancreatic regions were carefully inspected and palpated; no macroscopically enlarged or suspicious nodes were identified, and no lymph node biopsy was therefore taken. No cholangioscopy still images were retrievable from the archived intraoperative video. Estimated intraoperative blood loss was 300 mL, and no transfusion was required.

Gross specimens included a 10 × 6 × 6 cm right anterior section containing a firm, capsular-invasive white tumor in segment VIII (measuring 3.5 × 3.5 × 2 cm, with nearest parenchymal margin >1 cm) and a dilated bile duct containing mucus and papillary IPNB-like tissue contiguous with the tumor; a 3 × 3 × 2 cm segment VII specimen containing a soft brown mass measuring 3 × 2 × 2 cm without capsular invasion, with hemorrhage and necrosis and nearest parenchymal margin >1 cm (**Figure 3**).

**Figure 3:**
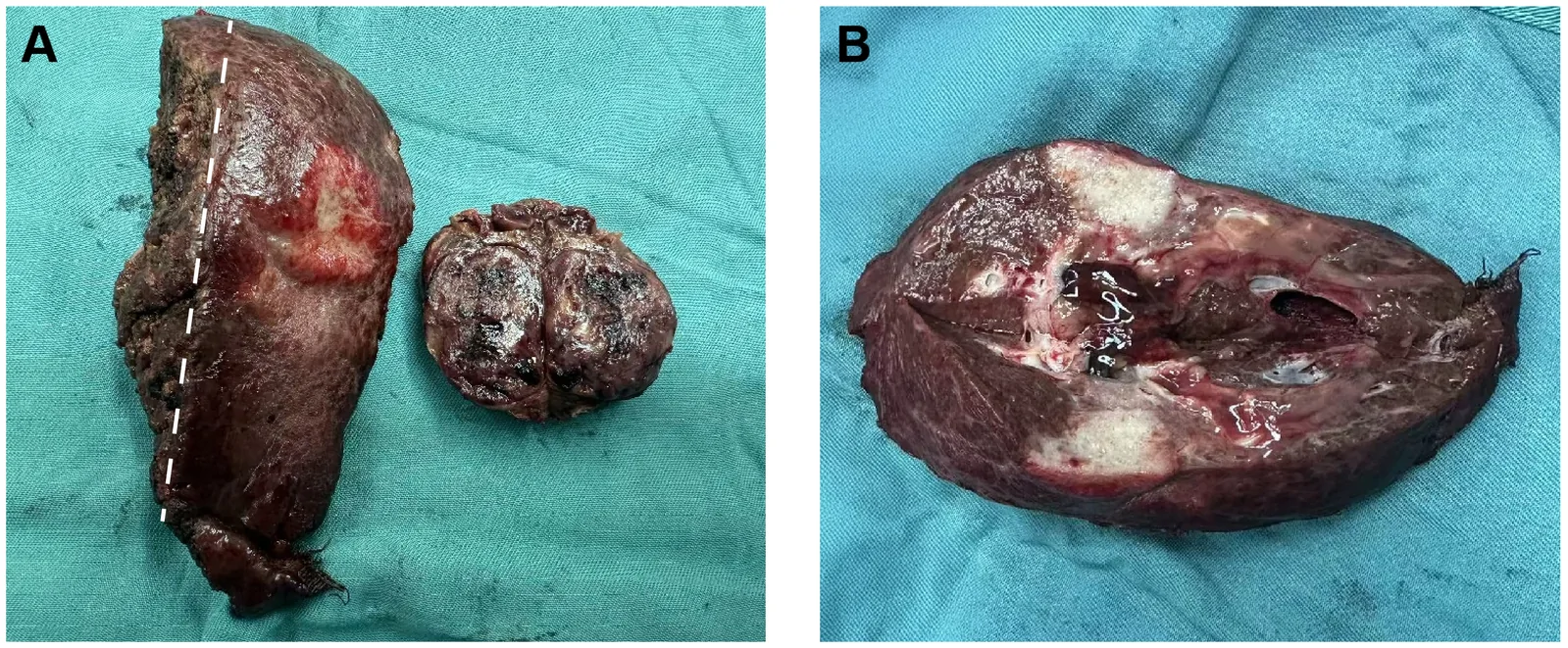
Gross surgical specimens. **(A)** Resected right anterior section (segments V and VIII; left) and segment VII specimen (right). The white dashed line marks the parenchymal resection margin of the right anterior section specimen. On gross pathological examination, the segment VIII tumor was firm and white, measured 3.5 × 3.5 × 2 cm, and showed capsular invasion; the segment VII tumor was soft and brown, measured 3 × 2 × 2 cm, and showed hemorrhage and necrosis without capsular invasion. The nearest parenchymal resection margin exceeded 1 cm for each tumor. **(B)** Opened right anterior section specimen. Gross examination demonstrated a dilated bile duct containing mucus and papillary IPNB-like tissue contiguous with the tumor (IPNB, intraductal papillary neoplasm of the bile duct).

Intraoperative frozen section revealed intrahepatic cholangiocarcinoma (ICC) in the right anterior section, hepatocellular carcinoma (HCC) in segment VII, and biliary IPNB with high-grade intraepithelial neoplasia, with negative proximal and distal bile duct margins. Final pathology confirmed moderately to poorly differentiated ICC (3.5 × 3.5 × 2 cm) in segment VIII, directly contiguous with a 0.8 cm high-grade intraductal papillary neoplasm of the bile duct (IPNB) protruding into the right hepatic duct, and well-differentiated HCC (3 × 2 × 2 cm) in segment VII. On step sections through the biliary lesion, high-grade intraductal papillary epithelium was directly contiguous with invasive ICC, with infiltrative glands in desmoplastic stroma at the base of the papillary lesion. **Figure 4D** illustrates the intraductal IPNB and invasive ICC components and their interface. Panels A–C show serial sections from one sampling site of the HCC, whereas panels D–F show serial sections from a separate sampling site of the biliary lesion; panels E and F depict higher-magnification immunohistochemical views corresponding to the boxed interface in panel D. This morphological continuity supports the diagnosis of IPNB-associated ICC. The background non-tumor liver showed nodular cirrhosis. Serial sectioning at multiple levels confirmed that the HCC and ICC components were separated by non-neoplastic parenchyma without transitional areas, thereby excluding combined hepatocellular-cholangiocarcinoma (cHCC-CCA). The IPNB was classified as Type 1 according to the Japan-Korea consensus scheme (7, 8), and as the pancreatobiliary subtype according to the WHO 2019 classification (9). Although Type 1 IPNBs in this scheme are more typically of intestinal or oncocytic subtype, the present lesion was assigned Type 1 based on its overall architectural pattern (cystic/fusiform dilatation with papillary proliferation and relatively low-grade architecture in non-invasive areas), while the lesion was morphologically consistent with the pancreatobiliary subtype based on features including complex papillary architecture with high-grade cellular atypia and focal mucin production. MUC1, MUC2, MUC5AC, MUC6, and CDX2 immunohistochemistry was not performed. No vascular invasion or tumor thrombus, lymphovascular invasion, or perineural invasion was identified. The hepatic parenchymal resection margins for both tumors exceeded 1 cm, and the proximal and distal bile duct margins were negative on frozen-section examination. No regional lymph nodes were submitted for histological examination; therefore, pathological nodal status was unassessed (pNx) for both tumors. According to the AJCC 8th edition, the ICC was staged as pT1a pNx cM0 (stage group indeterminate due to pNx), the HCC as pT1b pNx cM0 (stage group indeterminate due to pNx), and the non-invasive high-grade IPNB component as pTis. In the HCC, HepPar-1 and arginase-1 (Arg-1) showed diffuse cytoplasmic positivity (**Figures 4B, C**, respectively). In the biliary lesion, CK19 showed diffuse cytoplasmic positivity in both the high-grade IPNB and invasive ICC components (**Figure 4E**), whereas CK7 showed focal cytoplasmic positivity in the invasive ICC component (**Figure 4F**). Additional immunohistochemical findings included CK20 negativity and focal p53 expression (wild-type pattern) in the IPNB and ICC components, and focal GPC-3 positivity and glutamine synthetase positivity in the HCC. The Ki-67 proliferation index was 5% in the HCC. Taken together, the final diagnosis was synchronous well-differentiated HCC (segment VII) and IPNB-associated ICC (segment VIII) with adjacent high-grade IPNB of the right hepatic duct, arising in a patient with resolved HBV infection and HCV infection with SVR.

**Figure 4:**
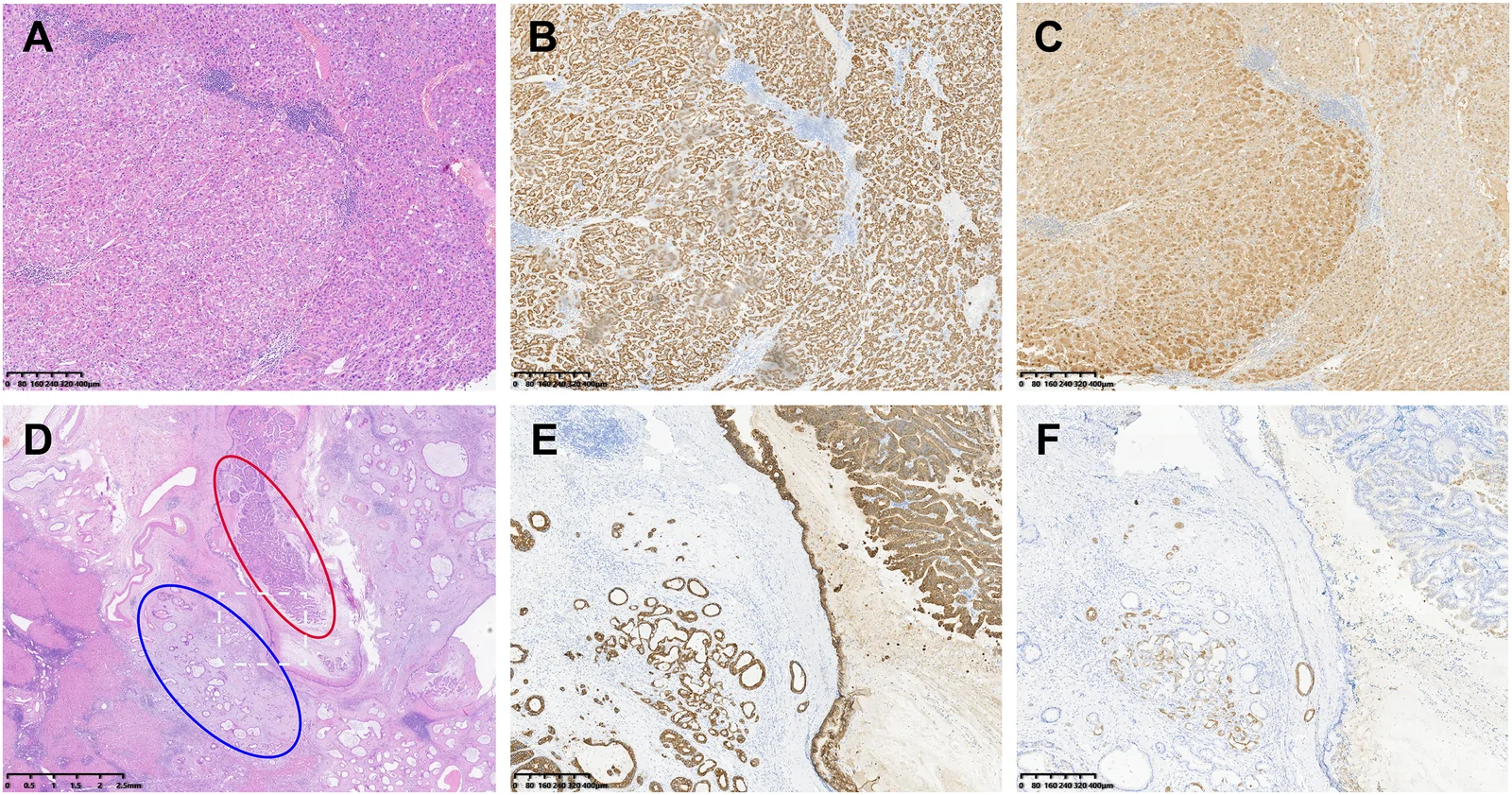
Histopathological and immunohistochemical findings. **(A–C)** Serial sections from the same sampling site of the segment VII hepatocellular carcinoma (HCC). **(A)** Well-differentiated HCC on hematoxylin and eosin (H&E) staining. **(B, C)** Diffuse cytoplasmic positivity for HepPar-1 **(B)** and arginase-1 (Arg-1; C) in HCC cells. **(D–F)** Serial sections from a separate sampling site of the segment VIII biliary lesion. **(D)** H&E-stained overview showing the non-invasive intraductal papillary neoplasm of the bile duct (IPNB) component with high-grade intraepithelial neoplasia (red ellipse) and associated moderately to poorly differentiated invasive intrahepatic cholangiocarcinoma (ICC; blue ellipse). The white dashed box marks the IPNB–ICC interface region shown in the CK19- and CK7-stained serial sections in E and F, respectively. **(E)** Diffuse cytoplasmic CK19 positivity in both the IPNB and invasive ICC components. **(F)** Focal cytoplasmic CK7 positivity in the invasive ICC component. Objective magnification: ×4 (A–C, E, F); ×1 **(D)**. Scale bars: 400 μm (A–C, E, F); 2.5 mm **(D)**.

The patient recovered uneventfully, with no complications of Clavien-Dindo grade II or higher (no biliary leak, hemorrhage, or infection). Adjuvant chemotherapy was not administered: following multidisciplinary tumor board (MDT) review and shared decision-making with the patient, adjuvant therapy was omitted after individualized consideration of his advanced age, postoperative recovery status, and the potential benefits and toxicity of treatment. At 3 months of follow-up, contrast-enhanced CT performed at 1 and 3 months postoperatively showed no evidence of recurrence. PIVKA-II normalized, while AFP, CA19-9, and CEA remained within normal limits. Planned surveillance comprises contrast-enhanced CT or MRI together with tumor marker assessment every 3 months during the first 2 postoperative years and every 6 months thereafter. The clinical timeline from completion of antiviral therapy through symptom onset, surgery, and postoperative follow-up is summarized in **Supplementary Figure 1**.

Patient Perspective: During the preoperative evaluation, the patient was informed of the preliminary diagnosis of metastatic cholangiocarcinoma and the possibility of unresectable disease. He expressed a strong preference for active surgical exploration after being counseled on the limitations of imaging-based diagnosis in rare synchronous neoplasms. Postoperatively, the patient reported significant relief of abdominal pain and pruritus within two weeks. At the 3-month follow-up, he remained satisfied with his functional recovery and understood the necessity of close surveillance despite the absence of radiological recurrence.

## Discussion

The preoperative misdiagnosis of multifocal hepatic lesions with a biliary mass as metastatic disease, rather than synchronous primary neoplasms, is a recognized diagnostic challenge, as illustrated by reports of synchronous double primary liver cancers being mistaken for metastatic malignancy (10–12). Accurate staging is paramount to stratifying patients appropriately for curative versus palliative therapy in hepatobiliary oncology (13, 14). In the absence of confirmed distant metastases, the synchronous occurrence of distinct primary hepatic neoplasms, although rare, should be entertained in the differential diagnosis (10–12). Laparoscopic exploration confirmed resectability and excluded peritoneal dissemination, consistent with the established role of staging laparoscopy in detecting radiologically occult metastatic disease (13, 15). Conversion to open surgery was necessitated by the hilar location of the biliary mass and the technical complexity of biliary reconstruction, in accordance with expert consensus recommendations that conversion to laparotomy is indicated when hilar exposure or biliary anastomosis proves technically infeasible under laparoscopy (16), with the technical demands of laparoscopic biliary-enteric reconstruction in hilar cholangiocarcinoma well documented (17). Intraoperative cholangioscopy provided direct visualization of the intraductal neoplasm (18, 19), as supported by studies demonstrating the diagnostic value of direct cholangioscopic visualization of the biliary tree (20), while frozen-section analysis confirmed negative bile duct margins and guided the extent of resection in this patient, consistent with established practice in perihilar cholangiocarcinoma surgery (21, 22).

In the present case, histopathology established the diagnoses of HCC and IPNB-associated ICC. Diffuse cytoplasmic HepPar-1 and arginase-1 positivity supported hepatocellular differentiation in the HCC, whereas CK19 positivity in both biliary components and focal CK7 positivity in the invasive ICC supported biliary differentiation (**Figure 4**) (23). Histological separation by non-neoplastic parenchyma without transitional areas, supported by lineage-specific immunophenotypes, excluded combined hepatocellular-cholangiocarcinoma (cHCC-CCA) (23, 24). Histological continuity between the high-grade IPNB and adjacent invasive ICC provided the principal morphological basis for a possible IPNB-to-ICC progression sequence (8, 25, 26); however, morphology alone does not establish the temporal or clonal relationship between these components. The shared CK19 expression supports biliary differentiation but does not establish a clonal relationship; the focal wild-type p53 staining provides no independent evidence of progression, and the reported Ki-67 index pertains to the separate HCC. According to the WHO 2019 classification, the IPNB components were classified as the pancreatobiliary subtype (7, 9, 27) and, under the type 1/type 2 subclassification, corresponded to type 1 (7, 9, 27). The ICC corresponded to pT1a pNx cM0 and the HCC to pT1b pNx cM0 according to the 8th edition of the AJCC TNM staging system (28, 29); stage groups remained indeterminate because regional lymph nodes were not histologically examined. Negative hepatic and bile duct margins established a margin-negative (R0) resection (7, 30), which is distinct from the completeness of pathological staging.

Synchronous HCC and ICC have been reviewed in a recent case series and literature review (1). To contextualize the rarity of our case, we performed a targeted literature review of PubMed on September 28, 2026. The main search concepts were IPNB, HCC, and ICC; the search query was: (“intraductal papillary neoplasm of the bile duct”[Title/Abstract] OR “IPNB”[Title/Abstract] OR “biliary papillomatosis”[Title/Abstract] OR “papillary cholangiocarcinoma”[Title/Abstract] OR “mucin-producing bile duct tumor”[Title/Abstract]) AND (“hepatocellular carcinoma”[Title/Abstract] OR “HCC”[Title/Abstract]) AND (“intrahepatic cholangiocarcinoma”[Title/Abstract] OR “ICC”[Title/Abstract] OR “cholangiocarcinoma”[Title/Abstract]). The reference lists of all retrieved articles were additionally hand-searched. This search retrieved 17 records. Studies were excluded if they described (i) IPNB or HCC alone without concurrent ICC; (ii) combined hepatocellular-cholangiocarcinoma (cHCC-CCA) rather than spatially separate primaries; or (iii) IPNB-associated ICC without coexistent HCC. In addition, a report of resected IPNB with synchronous HCC and pancreatic IPMN (31) was identified through the synonym-expanded search and was excluded under criterion (i), as no invasive ICC was documented in that patient. Only one prior report (2) met the criterion of documenting HCC, IPNB, and invasive IPNB-associated ICC in a single patient. That patient died of progressive hepatic failure after transarterial chemoembolization (TACE) without attempted resection (**Supplementary Table 1**) (2). Within the scope of this targeted review, the present case represents, to our knowledge, the first reported margin-negative (R0) resection for this specific entity. The association with resolved HBV and HCV-SVR offers a hypothesis-generating signal regarding viral hepatitis–related hepatobiliary malignancies, though any mechanistic link requires independent validation.

The decision not to administer adjuvant chemotherapy warrants discussion. The BILCAP trial demonstrated that adjuvant capecitabine improved overall survival in the per-protocol analysis of patients with resected biliary tract cancer (32), and current BSG guidelines recommend consideration of adjuvant capecitabine following R0 resection of cholangiocarcinoma (14). In this case, the MDT and the patient made a shared decision to omit adjuvant therapy after considering his advanced age (77 years), postoperative recovery status, and concerns about capecitabine toxicity in elderly patients, including hand-foot syndrome and diarrhea (33). The patient was counseled regarding the potential benefits and risks. Unassessed nodal status (pNx) does not establish node-negative disease or independently justify omission of adjuvant therapy.

## Limitations

This study is limited by the short three-month follow-up, which documents an early postoperative outcome and is insufficient to assess long-term recurrence or survival. Paired molecular analysis of the IPNB and ICC components was not performed; therefore, their clonal relationship and the proposed IPNB-to-ICC progression sequence remain unconfirmed. The absence of regional lymph node sampling (pNx) limits staging and prognostic assessment despite negative resection margins. The lack of suspicious nodes on imaging or intraoperative inspection does not exclude microscopic nodal metastases. Molecular confirmation of viral involvement—including *in situ* hybridisation for HBV DNA and HCV RNA, and testing for HBV cccDNA or integrated HBV DNA—was not performed on the resected tissue; any causal or contributory role of resolved HBV or HCV-SVR in this tumour complex therefore remains hypothesis-generating. Mucin and CDX2 immunohistochemistry (MUC1, MUC2, MUC5AC, MUC6, and CDX2) was not performed, limiting precise IPNB subtype assignment. Detailed histological characterization of the non-tumor liver tissue (fibrosis stage, steatosis, and inflammatory activity) could not be retrieved from the archived pathology records. The exact calendar date of the last negative preoperative HCV RNA test was unavailable for this report. Finally, this is a single-patient case report, and the findings are not generalizable.

## Conclusion

Three lessons emerge from this case. First, multifocal hepatic lesions with a biliary mass should not be reflexively interpreted as metastatic disease; in this case, intraoperative frozen-section analysis and cholangioscopy were useful for diagnosis and margin-negative resection. Second, histological continuity between high-grade IPNB and invasive ICC supports the diagnosis of IPNB-associated ICC and suggests a possible progression sequence; the immunophenotype supports biliary differentiation but does not establish a clonal relationship. Third, the association with resolved HBV infection and HCV infection with SVR offers a hypothesis-generating signal regarding viral hepatitis–related hepatobiliary malignancies. The 3-month follow-up represents only an early postoperative outcome, and longer-term surveillance is required to assess recurrence and survival.

## Data Availability

The raw data supporting the conclusions of this article will be made available by the authors, without undue reservation.

## Supplementary material

Supplementary Figure 1 Clinical timeline from completion of antiviral therapy approximately 3 years before admission through symptom onset, diagnosis, surgery, and postoperative follow-up at 1 and 3 months. Abbreviations: HCV, hepatitis C virus; SVR, sustained virological response; FLR, future liver remnant; ICGR15, indocyanine green retention rate at 15 minutes; PIVKA-II, protein induced by vitamin K absence or antagonist-II; AFP, alpha-fetoprotein; CA19-9, carbohydrate antigen 19-9; CEA, carcinoembryonic antigen; FU, follow-up.

Supplementary Table 1 Comparison of reported cases documenting concurrent HCC, IPNB, and invasive IPNB-associated ICC.
