# Supplementary material for: Synchronous hepatocellular carcinoma and IPNB-associated intrahepatic cholangiocarcinoma in a patient with resolved viral hepatitis: a case report of diagnostic pitfall and margin-negative resection
Source: Front Oncol. 2026 Oct 28;16:1894631. doi: 10.3389/fonc.2026.1894631 (PMC13751510; doi:10.3389/fonc.2026.1894631)
Supplement: Supplementary file 1 [file Table1.docx]

**Supplementary Table 1. Comparison of reported cases documenting concurrent HCC, IPNB, and invasive IPNB-associated ICC.**

| **Characteristic** | **Current Case** | **Previous Case (Xu et al., 2011)** |
| --- | --- | --- |
| **Age/Sex** | 77 years / Male | 76 years / Male |
| **Viral hepatitis background** | Resolved HBV (HBsAg-negative, anti-HBc/anti-HBe positive); HCV with SVR after sofosbuvir/velpatasvir | HBV-related cirrhosis (HBsAg-positive for 17 years, cirrhosis for ~10 years) |
| **Clinical presentation** | Intermittent RUQ pain, abdominal distension >6 months; acute exacerbation with scleral icterus, pruritus, nausea | Recurrent HCC in right lobe; admitted for TACE |
| **Tumor markers** | PIVKA-II elevated (52.3 ng/mL); AFP, CA19-9, CEA normal | AFP normal; CEA elevated (114.6 ng/mL); CA19-9 markedly elevated (52,000 U/mL) |
| **HCC component** | Well-differentiated HCC in segment VII (3 × 2 × 2 cm); nodular cirrhosis; no capsular invasion | Two nodules in right lobe (1.7 × 1.4 cm and 1.2 × 1.3 cm); post-TACE coagulative necrosis with trabecular growth pattern |
| **IPNB component** | Pedunculated IPNB arising from right hepatic duct; high-grade intraepithelial neoplasia; Type 1 (Nakanuma/Japan-Korea consensus); pancreatobiliary subtype (WHO 2019) | Exophytic papillary proliferation in dilated left intrahepatic large bile ducts; cystic/fusiform dilatation with mucin hypersecretion |
| **ICC component** | Moderately to poorly differentiated ICC in segment VIII (3.5 × 3.5 × 2 cm); direct continuity with adjacent high-grade IPNB | Invasive mucinous carcinoma in left lobe; associated with IPNB; Alcian blue and PAS-positive |
| **Immunohistochemistry** | ICC/IPNB: CK7 (focal+), CK19 (+), CK20 (−), p53 (focal, wild-type) HCC: HepPar-1 (+), GPC-3 (focal+), Arginase-1 (+), glutamine synthetase (+), Ki-67 5% | IPNB: CK7 (diffuse+), CK19 (diffuse+), MUC5AC (moderate+), CK20 (focal+), CDX2 (focal+), NCAM (focal+), MUC1 (focal+) Negative: MUC2, MUC6, HepPar-1, AFP |
| **Treatment** | Open margin-negative (R0) resection (right anterior sectionectomy (segments V and VIII) combined with segment VII segmentectomy); intraoperative cholangioscopy and frozen-section analysis; Pringle maneuver ×3 | TACE only; no surgical resection attempted |
| **Resection margins** | Hepatic parenchymal margins >1 cm; proximal and distal bile duct margins negative on frozen section | Not applicable |
| **Lymph node status** | Regional lymphadenectomy not performed (pNx) | Not applicable |
| **Staging (AJCC 8th)** | ICC: pT1a pNx cM0 (stage group indeterminate due to pNx); HCC: pT1b pNx cM0 (stage group indeterminate due to pNx); IPNB: pTis | Not applicable |
| **Adjuvant therapy** | None (shared decision after multidisciplinary tumor board review, considering advanced age, postoperative recovery status, and potential treatment toxicity) | None |
| **Hospital course** | Uneventful recovery; no biliary leak, hemorrhage, or infection | Died of progressive hepatic failure after 4-month hospitalization |
| **Follow-up/Outcome** | 3 months: No recurrence on CT; PIVKA-II normalized; AFP, CA19-9, CEA remained normal | Autopsy case; death from progressive hepatic failure |
| **Key pathological distinction** | HCC and ICC separated by non-neoplastic parenchyma without transitional zones; excluded cHCC-CCA | Extensive intraductal spread from reactive bile ductules to interlobular/septal bile ducts to large bile ducts; speculated hepatic progenitor cell origin |
